# Supplementary material for: Pathogenicity of Asymptomatically Residing Fusarium Species in Non-Gramineous Plants and Weeds to Spring Wheat under Greenhouse Conditions
Source: Pathogens. 2022 Dec 4;11(12):1467. doi: 10.3390/pathogens11121467 (PMC9785125; doi:10.3390/pathogens11121467)
Supplement: Supplementary file 1 [file pathogens-11-01467-s001.zip › pathogens-2026676-supplementary.pdf]

pathogens

MDPI

Supplementary Materials

Table S1. Treatments in Experiment I for assessing the pathogenicity of *Fusarium* spp. derived from non-gramineous plants to spring wheat under greenhouse conditions.

**Code**	***Fusarium* species**	**Hostplant (Latin name)**	**Plant tissue**
BN42s	*F. avenaceum*	Spring oilseed rape (*Brassica napus*)	Stem
BN19c	*F. avenaceum*	Spring oilseed rape (*Brassica napus*)	Crown
N49fl	*F. avenaceum*	Spring oilseed rape (*Brassica napus*)	Flower
PS3r	*F. avenaceum*	Field pea (*Pisum sativum*)	Root
PS10fl	*F. avenaceum*	Field pea (*Pisum sativum*)	Flower
PS27l	*F. avenaceum*	Field pea (*Pisum sativum*)	Leave
BV32.2l	*F. avenaceum*	Sugar beet (*Beta vulgaris*)	Leave
BV33.3s	*F. avenaceum*	Sugar beet (*Beta vulgaris*)	Stem
BV21.2l	*F. avenaceum*	Sugar beet (*Beta vulgaris*)	Leave
8SW4SP6	*F. avenaceum*	Spring wheat (*Triticum aestivum*)	Head
8SW2SP7	*F. avenaceum*	Spring wheat (*Triticum aestivum*)	Head
8SW4SP8	*F. avenaceum*	Spring wheat (*Triticum aestivum*)	Head
7BN2l5	*F. culmorum*	Winter oilseed rape (*Brassica napus*)	Leave
BN26r	*F. culmorum*	Spring oilseed rape (*Brassica napus*)	Root
BN39fl	*F. culmorum*	Spring oilseed rape (*Brassica napus*)	Flower
PS38r	*F. culmorum*	Field pea (*Pisum sativum*)	Root
PS333.r	*F. culmorum*	Field pea (*Pisum sativum*)	Root
PS23r	*F. culmorum*	Field pea (*Pisum sativum*)	Root
BV161.1pe	*F. culmorum*	sugar beet (*Beta vulgaris*)	Petiole
BV15.1l	*F. culmorum*	sugar beet (*Beta vulgaris*)	Leave
BV142.1pe	*F. culmorum*	Sugar beet (*Beta vulgaris*)	Petiole
8SW4SP11	*F. culmorum*	Spring wheat (*Triticum aestivum*)	Head
8SW5SP2	*F. culmorum*	Spring wheat (*Triticum aestivum*)	Head
8SW1SP3	*F. culmorum*	Spring wheat (*Triticum aestivum*)	Head
7BN2l1	*F. graminearum*	Winter oilseed rape (*Brassica napus*)	Leave
BN98c	*F. graminearum*	Winter oilseed rape (*Brassica napus*)	Crown
BN425l	*F. graminearum*	Spring oilseed rape (*Brassica napus*)	Leave
PS21.2l	*F. graminearum*	Field pea (*Pisum sativum*)	Leave
5PS1s2	*F. graminearum*	Field pea (*Pisum sativum*)	Stem
5PS3p3-1	*F. graminearum*	Field pea (*Pisum sativum*)	Pod
BV7L6	*F. graminearum*	Sugar beet (*Beta vulgaris*)	Leave
BV463.1l	*F. graminearum*	Sugar beet (*Beta vulgaris*)	Leave
BV7L13	*F. graminearum*	Sugar beet (*Beta vulgaris*)	Leave
6SW4SP1	*F. graminearum*	Spring wheat (*Triticum aestivum*)	Head
6SW5SP1	*F. graminearum*	Spring wheat (*Triticum aestivum*)	Head
6SW5SP19	*F. graminearum*	Spring wheat *Triticum aestivum*)	Head
BN9fl1	*F. sporotrichioides*	Spring oilseed rape *Brassica napus*)	Flower
BN11l	*F. sporotrichioides*	Spring oilseed rape (*Brassica napus*)	Leave
BN33r2	*F. sporotrichioides*	Spring oilseed rape (*Brassica napus*)	Root
PS12p	*F. sporotrichioides*	Field pea (*Pisum sativum*)	Pod
PS14fl	*F. sporotrichioides*	Field pea (*Pisum sativum*)	Flower
PS37s	*F. sporotrichioides*	Field pea (*Pisum sativum*)	Stem

BV33.2s	*F. sporotrichioides*	Sugar beet (*Beta vulgaris*)	Stem
BV50.1s	*F. sporotrichioides*	Sugar beet (*Beta vulgaris*)	Stem
9SWSP17	*F. sporotrichioides*	Spring wheat (*Triticum aestivum*)	Head
8SW5SP19	*F. sporotrichioides*	Spring wheat (*Triticum aestivum*)	Head
Control (sterile water)			

Table S2. Treatments in Experiment II for assessing the pathogenicity of *Fusarium* spp. isolated from weeds to spring wheat under greenhouse conditions.

**Code**	***Fusarium* species**	**Hostplant (Latin name)**	**Plant tissue**
TI1118c	*F. avenaceum*	Scentless Mayweed (*Tripleurospermum inodorum*)	Crown
TI1143s	*F. avenaceum*	Scentless Mayweed (*Tripleurospermum inodorum*)	Stem
VA1109s	*F. avenaceum*	European field pansy (*Viola arvensis*)	Stem
VA1110f	*F. avenaceum*	European field pansy (*Viola arvensis*)	Fruit
CBP1101fl	*F. avenaceum*	Shepherd's purse (*Capsella bursa-pastoris*)	Flower
CBP1149c	*F. avenaceum*	Shepherd's purse (*Capsella bursa-pastoris*)	Crown
PA1126s	*F. avenaceum*	Annual meadow grass (*Poa annua*)	Stem
PA1128f	*F. avenaceum*	Annual meadow grass (*Poa annua*)	Fruit
FC1178fl	*F. avenaceum*	Black bindweed (*Fallopia convolvulus*)	Flower
FC1180l	*F. avenaceum*	Black bindweed (*Fallopia convolvulus*)	Leave
8SWG1SP7	*F. avenaceum*	Spring wheat (*Triticum aestivum*)	Head
8SW4SP10	*F. avenaceum*	Spring wheat (*Triticum aestivum*)	Head
TI1115c	*F. culmorum*	Scentless Mayweed (*Tripleurospermum inodorum*)	Crown
TI1330r2	*F. culmorum*	Scentless Mayweed (*Tripleurospermum inodorum*)	Root
VA1105l	*F. culmorum*	European field pansy (*Viola arvensis*)	Leave
VA1164f	*F. culmorum*	European field pansy (*Viola arvensis*)	Fruit
CBP1147c	*F. culmorum*	Shepherd's purse (*Capsella bursa-pastoris*)	Crown
CBP1401r	*F. culmorum*	Shepherd's purse (*Capsella bursa-pastoris*)	Root
PA1129c	*F. culmorum*	Annual meadow grass (*Poa annua*)	Crown
PA1129f	*F. culmorum*	Annual meadow grass (*Poa annua*)	Fruit
FC1088r	*F. culmorum*	Black bindweed (*Fallopia convolvulus*)	Root
FC1180c	*F. culmorum*	Black bindweed (*Fallopia convolvulus*)	Crown
8SWTG5SP4	*F. culmorum*	Spring wheat (*Triticum aestivum*)	Head
8SWG1SP25	*F. culmorum*	Spring wheat (*Triticum aestivum*)	Head
TI1120c	*F. graminearum*	Scentless Mayweed (*Tripleurospermum inodorum*)	Crown
TI1265f	*F. graminearum*	Scentless Mayweed (*Tripleurospermum inodorum*)	Fruit
VA153l	*F. graminearum*	European field pansy (*Viola arvensis*)	Leave
VA541s	*F. graminearum*	European field pansy (*Viola arvensis*)	Stem
CBP1151f	*F. graminearum*	Shepherd's purse (*Capsella bursa-pastoris*)	Fruit
CBP1400l	*F. graminearum*	Shepherd's purse (*Capsella bursa-pastoris*)	Leave
PA1130c	*F. graminearum*	Annual meadow grass (*Poa annua*)	Crown
PA1350s	*F. graminearum*	Annual meadow grass (*Poa annua*)	Stem
FC144r	*F. graminearum*	Black bindweed (*Fallopia convolvulus*)	Root
FC544r	*F. graminearum*	Black bindweed (*Fallopia convolvulus*)	Root

6SW4SP1	*F. graminearum*	Spring wheat (*Triticum aestivum*)	Head
6SW5SP1	*F. graminearum*	Spring wheat (*Triticum aestivum*)	Head
TI1123s	*F. sporotrichioides*	Scentless Mayweed (*Tripleurospermum inodorum*)	Stem
TI1135l	*F. sporotrichioides*	Scentless Mayweed (*Tripleurospermum inodorum*)	Leave
VA1107f	*F. sporotrichioides*	European field pansy (*Viola arvensis*)	Fruit
VA1159f	*F. sporotrichioides*	European field pansy (*Viola arvensis*)	Fruit
CBP1101f	*F. sporotrichioides*	Shepherd's purse (*Capsella bursa-pastoris*)	Fruit
CBP1148f	*F. sporotrichioides*	Shepherd's purse (*Capsella bursa-pastoris*)	Fruit
FC1089c	*F. sporotrichioides*	Black bindweed (*Fallopia convolvulus*)	Crown
8SW5SP19	*F. sporotrichioides*	Spring wheat (*Triticum aestivum*)	Head
9SWSP17	*F. sporotrichioides*	Spring wheat (*Triticum aestivum*)	Head
Control (sterile water)			
